# Bioequivalence and Food Effect Assessment of 2 Fixed‐Dose Combination Formulations of Dolutegravir and Lamivudine

**DOI:** 10.1002/cpdd.740

**Published:** 2019-11-14

**Authors:** Teodora Pene Dumitrescu, Kavita Peddiraju, Caifeng Fu, Kalpana Bakshi, Shui Yu, Zhiping Zhang, Allan R. Tenorio, Chris Spancake, Shashidhar Joshi, Allen Wolstenholme, Kimberly Adkison

**Affiliations:** ^1^ Clinical Pharmacology Modeling and Simulation GlaxoSmithKline Collegeville Pennsylvania USA; ^2^ Pharma R&D GlaxoSmithKline Collegeville Pennsylvania USA; ^3^ PAREXEL International Durham North Carolina USA; ^4^ Clinical Development ViiV Healthcare Durham North Carolina USA; ^5^ Medicinal Science and Technology GlaxoSmithKline Collegeville Pennsylvania USA; ^6^ Biostatistics GlaxoSmithKline India Mumbai India; ^7^ Clinical Pharmacology ViiV Healthcare Durham North Carolina USA

**Keywords:** bioequivalence, dolutegravir, HIV, lamivudine

## Abstract

This single‐dose study evaluated the bioequivalence, food effect, and safety of 2 experimental, 2‐drug, fixed‐dose formulations of 50 mg dolutegravir and 300 mg lamivudine (formulation AH and formulation AK) as compared with coadministration of single‐entity tablets of 50 mg dolutegravir and 300 mg lamivudine (reference). In fasted subjects, formulation AH lamivudine exposure was similar to the reference; however, dolutegravir exposure was consistently higher in formulation AH, with area under the concentration‐time curve (AUC) and maximum concentration (C_max_) approximately 27% to 28% greater than reference. Formulation AK met bioequivalence standards to the reference for dolutegravir (AUC_0‐∞_ and C_max_) and lamivudine (AUC_0‐∞_ and AUC_0‐t_) exposure; however, dolutegravir AUC_0‐t_ and lamivudine C_max_ were approximately 16% and 32% higher than the reference, respectively. A high‐fat meal increased dolutegravir AUC and C_max_ by up to 33% and 21%, respectively, and decreased lamivudine C_max_ by approximately 30%. Both test and reference formulations were well tolerated. The results support further development of formulation AK as a novel, 2‐drug, fixed‐dose combination tablet treatment for patients with HIV.

The standard of care for patients infected with human immunodeficiency virus 1 (HIV‐1) is a life‐long regimen of 3 or more antiretroviral agents: 2 nucleoside reverse transcriptase inhibitors and a third drug from one of the following classes: protease inhibitors, integrase inhibitors, or nonnucleoside reverse transcriptase inhibitors.^1^ Multidrug regimens that require multiple tablets or capsules, especially with different dosing times, can suffer from decreased adherence and, consequently, reduced therapeutic efficacy.^2^, ^3^, ^4^ Contemporary regimens consist of fixed‐dose combinations of 2 or 3 drugs that are convenient to take, provide durable virologic suppression, and are relatively well tolerated. In addition, fixed‐dose combination tablets have been shown to enhance patient adherence to a life‐long treatment regimen and, consequently, to improve patient outcomes.^2^, ^4^, ^5^

Two drugs currently used in HIV treatment are dolutegravir and lamivudine. Dolutegravir is a potent HIV integrase strand transfer inhibitor^6^ with a high barrier for viral resistance^7^ that does not require pharmacokinetic boosting and can be administered once daily.^6^, ^8^, ^9^ Dolutegravir is rapidly absorbed when administered orally (time to peak concentration [t_max_] from 0.5 to 2 hours^10^) and has demonstrated dose proportionality for exposure (area under the concentration‐time curve [AUC]) over the range of 2 to 100 mg for a single dose oral suspension.^6^ In a mass balance study of 20 mg [^14^C]‐dolutegravir (80 µCi), unchanged dolutegravir was the predominant component in plasma, with elimination occurring in both feces and in urine (64.0% and 31.6% of dolutegravir recovered, respectively^11^). The primary metabolic pathway for dolutegravir is conversion to a pharmacologically inactive ether glucuronide by UDP‐glucuronosyltransferase (UGT) 1A1.^11^, ^12^ Other minor biotransformation pathways include oxidation by cytochrome P450 3A4 and metabolism by UGT1A3 and UGT1A9.^11^, ^12^ Because dolutegravir is often coadministered with other medications, it has been evaluated for drug‐drug interactions with more than 20 drugs, including other antiretrovirals, acid‐reducing agents, multivitamins, oral hormonal contraceptives, antimycobacterial agents, and drugs for treatment of hepatitis C (reviewed in Cottrell et al^13^) that required dose adjustment or dose staggering for etravirine, antacid‐containing magnesium and aluminum hydroxides, and rifampin. Dolutegravir also potently inhibits the organic cation transporter 2^12^ and multidrug and toxin extrusion 1 transporter.^14^ Based on in vitro organic cation transporter 2 and multidrug and toxin extrusion 1 inhibition, dolutegravir may increase plasma concentrations of drugs eliminated via these pathways, such as dofetilide and metformin, and a pharmacokinetic interaction with metformin has been demonstrated in a clinical study.^15^ Dolutegravir is also a substrate for P‐glycoprotein and breast cancer resistance protein in vitro.^12^

Lamivudine is a potent, well‐tolerated nucleoside reverse transcriptase inhibitor that can also be dosed once daily. Lamivudine has well‐established safety and pharmacokinetic profiles from over 2 decades of clinical use.^16^, ^17^, ^18^ Lamivudine is highly soluble and rapidly absorbed (t_max_ from 0.5 to 4.0 hours), with absolute bioavailability ranging from 82% to 86% for oral administration.^19^, ^20^ The majority of lamivudine is renally excreted, with approximately 70% of an administered oral dose eliminated as unchanged drug in urine over 24 hours.^21^ In particular, lamivudine is eliminated by filtration and active renal tubular secretion.^19^ Metabolism is a minor route of elimination, with only 5% to 10% of the parent drug metabolized to an inactive transsulfoxide metabolite that is excreted in the urine. In peripheral blood mononuclear cells, lamivudine is anabolized by phosphorylation to lamivudine triphosphate, the molecule required for antiviral activity.^18^ Lamivudine has demonstrated few interactions with cytochrome P450 enzymes, although drugs that are also renally excreted have the potential for interactions with lamivudine. Coadministered trimethoprim sulfamethoxazole increases lamivudine exposure (AUC_0‐∞_) by 43%; however, the increase is unlikely to result in toxicity based on the safety profile of higher lamivudine doses up to 300 mg administered twice daily.^22^ Coadministration with sorbitol decreases the absorption of lamivudine, with AUC_0‐∞_ being reduced by 14% to 36% and maximum concentration (C_max_) by 28% to 55%.^23^

Currently, dolutegravir and lamivudine are individually approved in the United States as Tivicay (ViiV Healthcare, Research Triangle Park, North Carolina) and Epivir (ViiV Healthcare), respectively. These drugs are also available as a single tablet fixed‐dose formulation with abacavir under the brand name Triumeq (ViiV Healthcare). Two large, multicenter, double‐blind, randomized, noninferiority studies in treatment‐naive, HIV‐infected adults (GEMINI‐1 and ‐2) recently evaluated dolutegravir and lamivudine as a 2‐drug regimen, with each coadministered as an individual tablet.^24^, ^25^ The GEMINI studies compared once‐daily coadministration of 50 mg dolutegravir plus 300 mg lamivudine to a standard of care, once‐daily, 3‐drug regimen consisting of a 50‐mg dolutegravir tablet plus a 2‐drug, fixed‐dose tablet containing 300 mg tenofovir disoproxil fumarate and 200 mg emtricitabine. The 2‐drug regimen was found to be noninferior to the 3‐drug regimen and was well tolerated.^24^, ^25^

To pursue new fixed‐dose tablet formulations, development was initiated for a 2‐drug single tablet containing 50 mg dolutegravir and 300 mg lamivudine. Two early formulations, formulations AA and AB, were developed, and the relative bioavailability assessed as part of a single‐dose, fasted, 3‐way crossover study with 6 treatment sequences (Study 204993, ClinicalTrials.gov Identifier NCT02738931; results published on the clinical trial registry^26^). These formulations (test) were compared with coadministration of individual 50‐mg dolutegravir and 300‐mg lamivudine tablets (reference). Both fixed‐dose combination formulations were safe and well tolerated. Exposure (based on AUC and C_max_) to dolutegravir in formulation AA was 12% to 14% greater than the reference, and lamivudine C_max_ was approximately 18% greater than the reference. For formulation AB, lamivudine C_max_ was approximately 23% greater than the reference. These data indicated a risk that the formulations (AA and AB) would not achieve bioequivalence to the coadministered individual components if entered into an appropriately powered study. Consequently, a modified version of formulation AA (formulation AH) and an alternative formulation approach (AK) were also developed, with each formulation again containing 50 mg dolutegravir and 300 mg lamivudine.

The current article summarizes the findings of a bioequivalence study comparing the experimental formulations AH and AK tablets (test) to coadministration of 50‐mg dolutegravir and 300‐mg lamivudine tablets (reference). Secondary objectives of the study were to determine safety and tolerability as well as to evaluate potential food effects.

## Methods

### Study Design and Treatment

The protocol for this study, Study 204994 (ClinicalTrials.gov Identifier NCT03078556), was approved by the Midlands Independent Review Board (Overland Park, Kansas). This study was conducted in accordance with Good Clinical Practice and the Declaration of Helsinki. All volunteers gave written informed consent. Study treatment was conducted at a single US site at Quintiles Phase One Services (Overland Park, Kansas).

**Figure 1:**
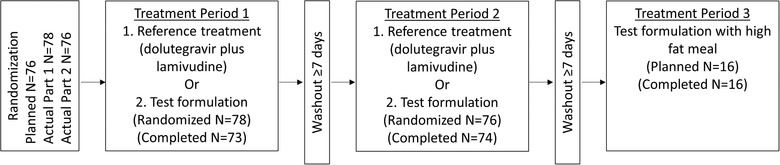
Study design for Study 204994 to assess the bioequivalence and food effect of 2 experimental formulations of fixed‐dose combination tablets (formulation AH, part 1, and AK, part 2) containing 50 mg dolutegravir and 300 mg lamivudine. Reference regimen administration was coadministration of individual, single‐entity tablets of 50 mg dolutegravir plus 300 mg lamivudine. Both parts of the study had equivalent designs: an open‐label, crossover design in which 76 subjects (planned) were randomized 1:1 to receive test or reference treatment in periods 1 and 2. In period 3, 16 subjects who had completed period 1 and period 2 also completed the food‐effect study, in which the test formulation was administered with a high‐fat meal. In part 1, subjects were administered formulation AH as the test drug. In part 2, a separate cohort of subjects received formulation AK.

This was a randomized, open‐label, 2‐part study, with 3 treatment periods per part, designed to evaluate the bioequivalence and food effect of 2 experimental fixed‐dose combination tablet formulations, formulation AH (part 1 test formulation) and formulation AK (part 2 test formulation). The study design is shown in Figure 1, with the design replicated for separate cohorts in part 1 and part 2. Comparison was to a reference treatment consisting of coadministration of 1 50‐mg dolutegravir tablet and 1 300‐mg lamivudine tablet.

The first 2 treatment periods (periods 1 and 2) in each part were randomized, crossover periods in which 76 subjects (randomized in a 1:1 ratio to test‐reference or reference‐test) received a single oral dose of the test or reference treatment. In periods 1 and 2, test and reference drugs were administered in the fasted state (after at least a 10‐hour fast) with 240 mL of room‐temperature water.

In the third treatment period, 16 subjects who had participated in and completed both periods 1 and 2 and who provided consent returned to receive a single dose of the fixed‐dose combination test formulation administered with a standard high‐fat meal as defined by the US Food and Drug Administration,^27^ which includes approximately 150, 250, and 500 to 600 dietary calories (kcal) from protein, carbohydrate, and fat, respectively.

Each period was separated by at least 7 days of washout.

### Investigational Products

The reference regimen included 1 tablet of dolutegravir (50 mg) and 1 tablet of lamivudine (300 mg). Dolutegravir tablets contained 50 mg dolutegravir, mannitol, microcrystalline cellulose, povidone, sodium starch glycolate, sodium stearyl, fumarate, and were coated with a poly(vinyl alcohol)‐based film coat. Lamivudine tablets were provided as commercially available lamivudine tablets and contained 300 mg lamivudine, microcrystalline cellulose, sodium starch glycolate, magnesium stearate, and were coated with a hydroxypropyl methylcellulose–based film coat.

Formulations AH and AK were 2‐drug, fixed‐dose, single‐tablet formulations, and both contained 50 mg dolutegravir, 300 mg lamivudine, mannitol, microcrystalline cellulose, povidone, sodium starch glycolate, sodium stearyl fumarate, and were coated with a hydroxypropyl methylcellulose–based film coat. Formulation AK additionally contained magnesium stearate.

### Study Population

Eligible subjects were healthy men and women between the ages of 18 and 55 years (inclusive) with a body mass index within the range of 18.5 to 31.0 kg/m^2^ (inclusive); men and women were at least 50 kg and 45 kg, respectively. Overall health was determined by medical history, physical examination, 12‐lead ECG, and laboratory tests. Women were enrolled only if they were of nonchildbearing potential or of childbearing potential, had a negative pregnancy test, and agreed to follow approved guidelines for contraception. Subjects had to possess the ability to swallow 2 tablets at the same time for administration of the reference treatment.

Subjects were excluded from the study if they had alanine aminotransferase (ALT) or bilirubin >1.5 times the upper limit of normal (ALT normal range, 21 to 72 IU/L; bilirubin normal range, 3.42 to 22.23 µmol/L); had a chronic history of liver disease or known hepatic/biliary abnormalities (except for Gilbert syndrome or asymptomatic gallstones); had a QT interval corrected for heart rate according to the Fredericia formula (QTcF) >450 ms; had a history of regular alcohol consumption (>14 drinks/wk for men and >7 drinks/wk for women) within 6 months of the study; had creatinine clearance <60 mL/min, indicating renal impairment; tested positive for either HIV, hepatitis B or hepatitis C virus; were current illicit drug users or had a positive prestudy drug/alcohol screen; were unable to refrain from the use of prescription or nonprescription drugs; had urinary cotinine levels indicative of smoking or a history or regular use of tobacco‐ or nicotine‐containing products within 1 month before screening; or had a history of sensitivity to the study medications.

### Sample Size Calculation

A sample size of 70 subjects was calculated to provide a 90% power to demonstrate bioequivalence for the fixed‐dose combination formulation compared with the reference treatment. The estimates of likely intersubject variability (coefficients of within‐subject variation, CV_w_%) and true geometric least‐squares (GLS) mean ratios were based on study 204993, which evaluated the relative bioavailability of early‐stage fixed‐dose formulations.^26^ The sample size calculation assumed a true geometric means ratio of 1.10 and within‐subject variability (CV_w_%) of 26% from study 204993 (data not published), log‐normal distribution of the data, and that each 1‐sided t‐test is made at the 5% level. These values were assumed based on results for dolutegravir pharmacokinetic parameters C_max_, AUC_0‐t_, and AUC _0‐∞_, which represented the highest CV_w_% (26%) when compared with those for lamivudine (CV_w_% of 9.0% to 20%).

Overall, the study enrolled 76 subjects such that a minimum of 70 evaluable subjects would complete treatment periods 1 and 2. Additional subjects were enrolled to account for subject attrition and unevaluable pharmacokinetic parameters.

### Pharmacokinetic Sampling and Bioanalytical Methods

Blood samples for pharmacokinetic sampling were taken predose and at 0.25, 0.5, 0.75, 1, 1.5, 2, 2.5, 3, 3.5, 4, 5, 6, 8, 12, 16, 24, 48, and 72 hours postdose for all periods. Plasma samples were analyzed for dolutegravir and lamivudine concentrations by Pharmaceutical Product Development (PPD; Middleton, Wisconsin) using a validated analytical method.

Dolutegravir was extracted from plasma using protein precipitation with acetonitrile containing the stable isotope‐labeled dolutegravir internal standard (GSK1349572‐d_7_‐^15^N), followed by analysis with ultraperformance liquid chromatography (Acquity UPLC, Waters, Milford, Massachusetts) tandem mass spectrometry using isocratic elution with 60% of 0.1% formic acid in water and 40% of 0.1% formic acid in acetonitrile on a Waters XBridge BEH C18 column (130 Å, 3.5 µm, 2.1 × 50 mm; room temperature; flow rate 0.475 mL/min for 1.50 minutes) and using an API 4000 mass spectrometer (SCIEX, Framingham, Massachusetts) with a turbo ion spray interface in positive ion electrospray mode. The lower and upper limits of quantification for dolutegravir were 20 ng/mL and 20000 ng/mL, respectively, using a 25‐µL aliquot of human plasma (K_2_EDTA anticoagulant). Precision and accuracy were evaluated as described in Song et al.^28^ Mass spectrometry was set with multiple reaction monitoring (m/z 428.02 to m/z 277.10 for the internal standard and m/z 420.00 to m/z 283.10 for dolutegravir).

Lamivudine and the internal standard [^13^C,^15^N_2_]‐lamivudine were extracted from plasma using protein precipitation, followed by analysis with high‐performance liquid chromatography using a Thermo Scientific BetaSil Silica 100 column [100 Å, 5 µm, 3.0 × 50 mm] and an LC‐30AD liquid chromatography pump (Shimadzu, Kyoto, Japan) with binary mobile‐phase gradient elution at room temperature (Mobile Phase A, 10 mmol/L ammonium formate with 0.1% formic acid in water; and Mobile Phase B, 0.1% formic acid in acetonitrile) and a stepwise flow rate of 0.65 mL/min to 1 mL/min. The initial mobile phase condition of 10:90 (A:B) at 0.65 mL/min was held for 1.7 minutes, adjusted to 50:50 at 1.0 mL/min from 1.8 to 2.8 minutes, held at 10:90 and 0.65 mL/min for 1 minute, and finally returned to 10:90 at 0.65 mL/min (total run time of 4 minutes per sample). Detection was performed using an API 3000 mass spectrometer (SCIEX, Framingham, Massachusetts) with triple quadrupole mass spectrometry and a turbo ion spray interface in positive ion electrospray mode. The assay had lower and upper limits of quantification of 2.5 ng/mL and 2500 ng/mL, respectively, using a 50‐µL aliquot of K_2_EDTA plasma. Precision and accuracy were evaluated using quality control samples as described by Adkison et al.^23^ Mass spectrometry was set with multiple reaction monitoring (m/z 233.0 for the internal standard and m/z 230.0 for lamivudine).

### Pharmacokinetic Analyses

The pharmacokinetic plasma concentration population included all subjects who underwent plasma pharmacokinetic sampling and had evaluable dolutegravir or lamivudine concentration results sufficient to calculate pharmacokinetic parameters. The pharmacokinetic parameter bioequivalence summary population included subjects who had evaluable pharmacokinetic parameters for both analytes for both periods 1 and 2. The pharmacokinetic parameter food summary population included all subjects who had evaluable pharmacokinetic parameters for both the fed and fasted states for their corresponding test formulation (AH or AK). Data from subjects who vomited within 4 hours of study drug administration were not considered as evaluable.

Separate pharmacokinetic parameters for dolutegravir and lamivudine were derived using noncompartmental analysis methods and actual sampling times with Phoenix WinNonlin v6.3 (Certara, LP, Princeton, New Jersey). From the plasma concentration‐time data, the pharmacokinetic parameters determined for each of the 2 analytes included C_max_, t_max_, AUC_0–t_, AUC_0‐∞_, AUC_0‐24_, half‐life (t_½_), apparent clearance, and lag time. AUC_0‐∞_, AUC_0‐t_, and C_max_ were used as primary end points for bioequivalence.

### Statistical Analyses

SAS version 9.4 (SAS Institute, Cary, North Carolina) was used for statistical analysis. Pharmacokinetic parameters for dolutegravir and lamivudine (except for t_max_ and lag time) were log_e_‐transformed and analyzed separately using a mixed‐effects model with fixed‐effect terms for period and treatment for each treatment comparison for the analysis of bioequivalence. For the analysis of food effect, the model included a fixed‐effect term for treatment (fed versus fasted). Subject was treated as a random effect in the model. Point estimates and their associated 90%CIs were constructed for the differences in pharmacokinetic parameter values between test and reference treatments. The point estimates and their associated 90%CIs were then back‐transformed to provide point estimates and 90%CIs for the ratios of pharmacokinetic parameters from test and reference treatments. The null hypothesis was that the true ratio of the geometric mean of the test treatment to the geometric mean of the reference treatment, gµ(test)/gµ(reference), for each primary pharmacokinetic end point, is either less than 0.800 or greater than 1.250. The alternative hypothesis was that the true ratio of the test treatment geometric mean to the reference treatment geometric mean is greater than or equal to 0.800 and less than or equal to 1.250. For each pharmacokinetic parameter designated as a primary end point, 2 1‐sided t‐test procedures^29^ with α = 0.05 for each 1‐sided test were used for hypothesis testing.

During statistical analysis, AUC_0‐t_ ad hoc sensitivity analyses were conducted by excluding subjects who had within‐subject differences in t_last_ between treatments resulting in different time windows for AUC_0‐t_ calculation for the test and reference formulations. Differences in test/reference AUC time windows could contribute to a bias in the AUC_0‐t_ geometric mean ratio and influence bioequivalence determination.^30^ Where applicable, results are presented with and without sensitivity analyses. Presented adjusted geometric mean values for pharmacokinetic parameters were the antilog of the adjusted mean (ls‐mean) of log transformed values, where ls‐mean can be defined as a linear combination (sum) of the estimated effects from a linear model.

### Safety Assessments

Safety assessments included evaluations for adverse events (AEs), pregnancy, physical examinations, vital signs (temperature, systolic and diastolic blood pressure, and pulse rate), ECGs, clinical laboratory assessments (hematology, clinical chemistry, urinalysis, HIV/hepatitis B and C, creatinine clearance, and follicle‐stimulating hormone). The safety population was comprised of all subjects who were enrolled in the study and who received at least 1 dose of the study drug.

## Results

### Subject Disposition and Demographics

A total of 154 subjects (78 subjects in part 1 and 76 subjects in part 2) were enrolled and randomized, with 147 completing the study (73 subjects in part 1 and 74 subjects in part 2). Sixteen subjects who completed treatment periods 1 and 2 and provided consent continued and participated in the food effect portion of the study. Approximately two thirds of the subjects enrolled in the study were male, and the majority were white (65%) and not Hispanic or Latino (90%). The mean age of the study population was 30.5 ± 10.3 years (range 18 to 55 years), with an overall mean body mass index of 25.7 ± 3.3 kg/m^2^. The demographics were comparable between subjects in part 1 and part 2 (Table 1).

**Table 1:** Disposition and Demographics for Study Subjects in Part 1, Part 2, and Overall

Parameter	Part 1	Part 2	Total
Subject disposition			
Number of subjects	78	76	154
Number of subjects completed as planned, n (%)	73 (94%)	74 (97%)	147 (95%)
Number of subjects withdrawn (any reason), n (%)	5 (6%)	2 (3%)	7 (5%)
Reasons for subject withdrawal, n (%)			
Lost to follow‐up	2 (3%)	1 (1%)	3 (2%)
Adverse events	2 (3%)	1 (1%)	3 (2%)
Physician decision^a^	1 (1%)	0	1 (<1%)
Subject demographics			
Age, y, mean (SD)	29.4 (9.37)	31.6 (11.18)	30.5 (10.33)
Sex, n (%)			
Female	25 (32%)	26 (34%)	51 (33%)
Male	53 (68%)	50 (66%)	103 (67%)
BMI, kg/m^2^, mean (SD)	25.51 (3.204)	25.83 (3.354)	25.67 (3.272)
Height, cm, mean (SD)	171.97 (8.381)	172.05 (8.592)	172.01 (8.458)
Weight, kg, mean (SD)	75.69 (12.476)	76.57 (12.122)	76.12 (12.270)
Ethnicity, n (%)			
Hispanic or Latino	11 (14%)	4 (5%)	15 (10%)
Not Hispanic or Latino	67 (86%)	72 (95%)	139 (90%)
Race, n (%)			
Black or African American	20 (26%)	23 (30%)	43 (28%)
American Indian or Alaskan Native	6 (8%)	1 (1%)	7 (5%)
Asian, East Asian Heritage	0	1 (1%)	1 (<1%)
Asian, Central/South Asian Heritage	1 (1%)	1 (1%)	2 (1%)
Asian, Southeast Asian Heritage	1 (1%)	0	1(<1%)
White, White/European Heritage	50 (64%)	50 (66%)	100 (65%)

BMI indicates body mass index.

a One subject was removed from the study due to elevated alanine aminotransferase and aspartate aminotransferase associated with alcohol use (subject had a positive alcohol breath test).

### Pharmacokinetics and Bioequivalence

**Figure 2:**
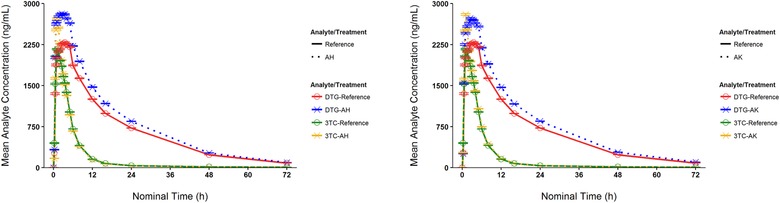
Arithmetic mean (standard error of mean) plasma concentration‐time curves in the fasted state for the analytes dolutegravir and lamivudine after administration of formulation AH or reference (left) and formulation AK or reference (right), where the reference was coadministration of dolutegravir and lamivudine. Solid lines are given for the reference treatment, and dotted lines are given for formulation AH or AK. AH and AK respectively indicate formulations AH and AK; DTG, dolutegravir; 3TC, lamivudine.

The dolutegravir and lamivudine mean concentration‐time profiles for formulations AH and AK compared with the reference are shown in Figure 2. The statistical comparison of plasma dolutegravir and lamivudine for experimental formulation AH or formulation AK versus coadministration of dolutegravir plus lamivudine is given in Table 2. For formulation AH, dolutegravir plasma AUC_0‐∞_, AUC_0‐t,_ and C_max_ were 27% to 28% higher than the reference. This observation was similar to the increased dolutegravir exposure noted with fixed‐dose combination formulations evaluated in a previous study (Study 204993). Therefore, formulation AH did not meet bioequivalence criteria for dolutegravir exposure. Exposure parameters (C_max_ and AUC) for lamivudine met established bioequivalence criteria, with the 90%CI of the GLS mean ratio falling between 0.80 and 1.25.

**Table 2:** Statistical Comparison of Plasma Dolutegravir and Lamivudine PK Parameters for Bioequivalence in the Fasted State

		Formulation AH (Part 1)			Formulation AK (Part 2)		
Parameter	Statistic	Test (n = 73)	Reference (n = 73)	GLS Mean Ratio (90%CI of the Ratio) [%CV_w_]	Test (n = 74)	Reference (n = 74)	GLS Mean Ratio (90%CI of the Ratio) [%CV_w_]
**Dolutegravir**							
AUC_0‐∞_, h·µg/mL	Mean (SD)	57.395 (16.654)	46.156 (16.753)	…	57.236 (17.860)	50.755 (19.188)	…
	Adjusted geomean	54.858	43.162	1.2710 (1.1894, 1.3582) [24.4]	54.559	47.239	1.1550 (1.0699, 1.2468) [28.5]
AUC_0‐t_, h·µg/mL	Mean (SD)	55.236 (15.836)	44.301 (15.956)	…	54.802 (16.734)	48.441 (17.890)	…
	Adjusted geomean	52.856	41.436	1.2756 (1.1919, 1.3651) [25.0]	52.337	45.204	1.1578 (1.0718, 1.2507) [28.7]
	Adjusted geomean	53.588^a^	43.207^a^	1.2403 (1.1604, 1.3257) [23.6]^a^	52.353^b^	46.109^b^	1.1354 (1.0527, 1.2246) [27.7]^b^
AUC_0‐24_, h·µg/mL	Mean (SD)	39.224 (11.198)	31.364 (10.921)	…	38.269 (11.376)	33.611 (11.694)	…
	Adjusted geomean	37.600	29.435	1.2774 (1.1931, 1.3676) [25.1]	36.613	31.566	1.1599 (1.0711, 1.2560) [29.7]
C_max_, µg/mL	Mean (SD)	3.231 (1.007)	2.566 (0.869)	…	3.040 (0.871)	2.703 (0.868)	…
	Adjusted geomean	3.082	2.407	1.2805 (1.1890, 1.3790) [27.4]	2.913	2.553	1.1410 (1.0533, 1.2361) [29.9]
CL/F, L/h	Mean (SD)	0.956 (0.312)	1.248 (0.517)	…	0.962 (0.305)	1.142 (0.465)	…
	Adjusted geomean	0.911	1.158	0.7868 (0.7363, 0.8408) [24.4]	1.058	0.916	0.8658 (0.8021, 0.9347) [28.5]
t_max_, h	Median	2.002 (0.500, 5.012)	2.007 (0.500, 8.009)	–0.127 (–0.500, 0.248)^f^	2.500 (0.500, 6.001)	2.501 (0.500, 5.011)	–0.127 (–0.497, 0.132)
t_½_, h	Mean (SD)	14.884 (2.314)	14.906 (2.351)	…	15.228 (2.757)	15.373 (2.737)	…
	Adjusted geomean	14.713	14.730	0.9988 (0.9829,1.0150) [5.8]	15.138	14.985	0.9899 (0.9760,1.0040) [5.2]
**Lamivudine**							
AUC_0‐∞_, h·µg/mL	Mean (SD)	13.004 (2.562)	12.572 (2.490)	…	13.773 (2.430)	12.985 (2.365)^c^	…
	Adjusted geomean	12.757	12.337	1.0341 (1.0097, 1.0591) [8.8]	13.588^c^	12.771^c^	1.0638 (1.0416, 1.0865) [7.7]
AUC_0‐24_, h·µg/mL	Mean (SD)	12.178 (2.451)	11.637 (2.400)	…	12.790 (2.340)	11.870 (2.331)	…
	Adjusted geomean	11.940	11.398	1.0475 (1.0185, 1.0773) [10.2]	12.581	11.642	1.0807 (1.0539,1.1081) [9.2]
AUC_0‐t_, h·µg/mL	Mean (SD)	12.855 (2.535)	12.398 (2.479)	…	13.567 (2.425)	12.700 (2.386)	…
	Adjusted geomean	12.612	12.160	1.0372 (1.0116, 1.0634) [9.1]	13.355	12.479	1.0702 (1.0464, 1.0946) [8.2]
	Adjusted geomean	12.685^d^	12.253^d^	1.0353 (1.0094, 1.0618) [9.0]^d^	13.409^e^	12.526^e^	1.0705 (1.0460, 1.0956) [8.3]^e^
C_max_, µg/mL	Mean (SD)	3.308 (0.898)	2.773 (0.808)	…	3.350 (0.951)	2.534 (0.677)	…
	Adjusted geomean	3.187	2.666	1.1956 (1.1437, 1.2498) [16.2]	3.219	2.443	1.3176 (1.2616, 1.3760) [15.9]
CL/F, L/h	Mean (SD)	23.959 (4.732)	24.793 (4.915)	…	22.475 (4.166)	23.896 (4.585)^c^	…
	Adjusted geomean	23.516	24.318	0.9670 (0.9442, 0.9904) [8.7]	22.080	23.489	0.9400 (0.9204, 0.9601) [7.7]
t_max_, h	Median	1.001 (0.500, 3.500)	1.005 (0.500, 4.005)	–0.126 (–0.253, –0.01)^f^	1.001 (0.500, 3.501)	1.006 (0.500, 4.002)	–0.248 (–0.376, –.001)^f^
t_½_, h	Mean (SD)	18.144 (4.659)	18.252 (5.273)	…	19.531 (6.078)	20.084 (6.735)^c^	…
	Adjusted geomean	17.646	17.585	1.0035 (0.9536, 1.0559) [18.6]	18.621	19.210	0.9693 (0.9214, 1.0198) [18.6]

AUC indicates area under the concentration‐time curve; CL/F, apparent clearance; C_max_, peak concentration; GLS, geometric least squares; PK, pharmacokinetic; %CV_w_, within‐subject coefficient of variation expressed as a percentage; t_max_, time to C_max_; t_½_, half‐time of elimination.

Note: The adjusted geometric mean is the antilog of the adjusted mean (ls‐mean) of log‐transformed values, where ls‐mean can be defined as a linear combination (sum) of the estimated effects from a linear model. GLS mean ratio calculated as test (experimental formulation) vs reference (coadministration of 50 mg dolutegravir plus 300 mg lamivudine).

a Five subjects had within‐subject differences in dolutegravir t_last_ between treatments. Four subjects had t_last_ of 48 hours in the reference treatment arm and t_last_ of 72 hours in the test arm; 1 subject had t_last_ of 48 hours in the test arm and t_last_ of 72 hours in the reference arm. An ad hoc sensitivity analysis was conducted by excluding these subjects from analysis (n = 68).

b Two subjects had within‐subject differences in dolutegravir t_last_ between treatments (t_last_ of 48 hours in the reference treatment arm and t_last_ of 72 hours in the test arm). An ad hoc sensitivity analysis was conducted by excluding these subjects from analysis (n = 72).

c One subject was excluded due to a result not being determined for the reference treatment in part 2 because this subject had a time interval for determination of t_½_ <2× the estimated t_½_ and the percentage AUC extrapolated was >20% (n = 73). Geometric mean analysis and bioequivalence analysis are reported with this subject's data excluded for the test formulation.

d Three subjects had between‐treatment differences in lamivudine t_last_ (t_last_ of 48 hours in the reference treatment arm and t_last_ of 72 hours in the test arm). An ad hoc sensitivity analysis was conducted by excluding these subjects from analysis (n = 70).

e Two subjects had between‐treatment differences in lamivudine t_last_. One subject had t_last_ of 48 hours in the reference treatment arm and t_last_ of 72 hours in the test arm; the other subject had t_last_ of 48 hours in the test arm and t_last_ of 72 hours in the reference arm. An ad hoc sensitivity analysis was conducted by excluding these subjects from analysis (n = 72).

f t_max_ statistical comparison is estimated median difference, calculated as Test – Reference (90%CI for the difference).

For formulation AK, dolutegravir met the established GLS mean ratio bioequivalence criteria for AUC_0‐∞_ and C_max_. The AUC_0‐t_ value for dolutegravir for formulation AK was ∼16% higher than that of the reference, with the upper limit of the 90%CI slightly above 1.25 (1.2507). However, 2 subjects had between‐treatment differences in t_last_ (48 hours versus 72 hours) that resulted in a different time window for the calculation of AUC_0‐t_. When these 2 subjects were excluded in an ad hoc sensitivity analysis, the dolutegravir AUC_0‐t_ GLS means ratio met bioequivalence criteria, with a ratio (90%CI) of 1.1354 (1.0527, 1.2246). AUC end points for lamivudine (AUC_0‐∞_ and AUC_0‐t_) met established bioequivalence criteria on initial analysis and after sensitivity analysis. Lamivudine C_max_ was approximately 32% higher than the reference.

One subject was excluded from the calculation of lamivudine AUC_0‐∞,_ clearance, and t_½_ for the test formulation (AK) in part 2, as the λz time duration was less than twice the calculated t_½_, and more than 20% of AUC_0‐∞_ was extrapolated. This subject was also excluded from the adjusted geometric mean analysis and subsequently excluded from the bioequivalence analysis for lamivudine AUC_0‐∞_.

### Food Effect

**Figure 3:**
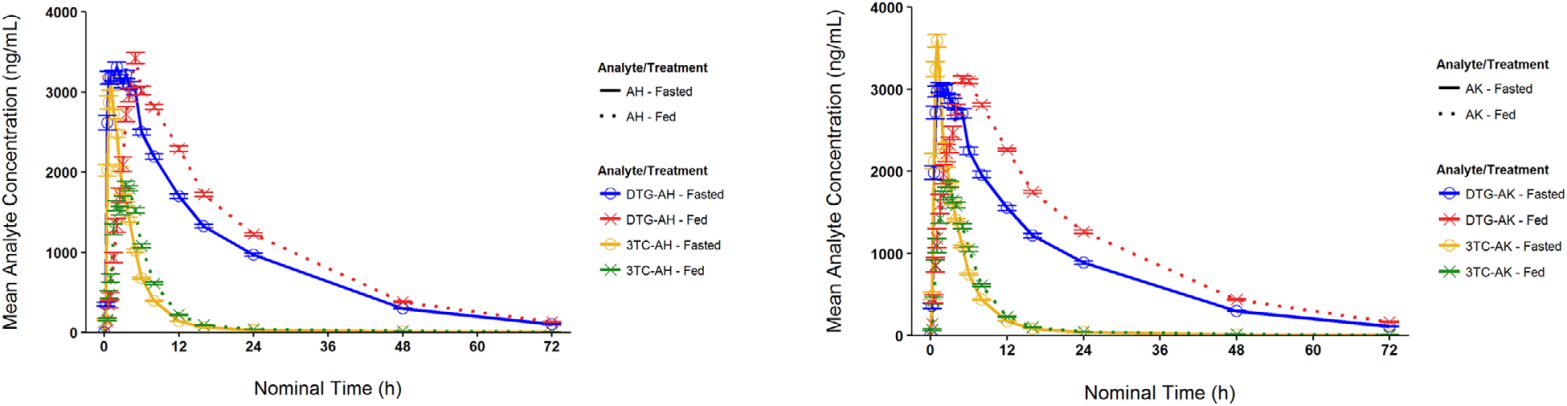
Arithmetic mean (standard error of mean) plasma concentration‐time curves for the analytes dolutegravir and lamivudine after administration of formulation AH (left) or formulation AK (right) in the fed and fasted states. Solid lines are given for the treatment administered in the fasted state, and dotted lines are given for treatment administered in the fed state. AH and AK respectively indicate formulations AH and AK; DTG, dolutegravir; 3TC, lamivudine.

The dolutegravir and lamivudine mean concentration‐time profiles in fed and fasted status for formulations AH and AK are shown in Figure 3. Pharmacokinetic parameters for experimental formulations given to subjects after at least a 10‐hour fast (fasted) or following consumption of a standard high‐fat meal (fed) are provided in Table 3. For formulation AH, a high‐fat meal resulted in an ∼15% increase in dolutegravir AUC_0‐∞_ and AUC_0‐t_ values and an ∼30% decrease in lamivudine C_max_. For formulation AK, the high‐fat meal resulted in an ∼32% increase in dolutegravir AUC_0‐∞_ and AUC_0‐t_, an ∼21% increase in dolutegravir C_max_, and an ∼30% decrease in lamivudine C_max_. For both formulations, dolutegravir t_max_ was prolonged from 1.5 to 5 hours. Lamivudine t_max_ was prolonged from 1 to 3.5 hours for formulation AH and from 1 to 2.75 hours for formulation AK.

**Table 3:** Statistical Comparison of Plasma Dolutegravir and Lamivudine PK Parameters for Food Effect Assessment

		Formulation AH (Part 1)			Formulation AK (Part 2)		
Parameter	Statistic	Fasted (n = 16)^a^	Fed (n = 16)	GLS Mean Ratio (90%CI) [%CV_w_]	Fasted (n = 16)	Fed (n = 16)	GLS Mean Ratio (90%CI) [%CV_w_]
**Dolutegravir**							
AUC_0‐∞_, h·µg/mL	Mean (SD)	57.363 (16.522)	73.310 (14.520)	**…**	60.907 (20.104)	78.189 (17.264)	**…**
	Adjusted geomean	62.344	71.978	1.1545 (1.0208, 1.3058) [20.1]	57.656	76.428	1.3256 (1.1837, 1.4845) [18.4]
AUC_0‐t_, h·µg/mL	Mean (SD)	55.217 (15.701)	70.402 (13.155)	**…**	58.232 (18.949)	74.125 (14.768)	**…**
	Adjusted geomean	60.321	69.256	1.1481 (1.0154, 1.2982) [20.0]	55.218	72.755	1.3176 (1.1750, 1.4775) [18.6]
C_max_, µg/mL	Mean (SD)	3.228 (0.996)	3.866 (0.779)	**…**	3.270 (1.045)	3.830 (0.804)	**…**
	Adjusted geomean	3.507	3.790	1.0808 (0.9527, 1.2261) [20.6]	3.101	3.752	1.2096 (1.0521, 1.3908) [22.8]
CL/F, L/h	Mean (SD)	0.955 (0.308)	0.708 (0.140)	**…**	0.919 (0.326)	0.669 (0.148)	**…**
	Adjusted geomean	0.802	0.695	0.8661 (0.7658, 0.9796) [20.1]	0.867	0.654	0.7544 (0.6736, 0.8448) [18.4]
t_max,_ h	Median (Min,Max)	1.50 (0.503, 5.00)	5.00 (2.00, 12.0)	3.017 (1.872, 4.496)^b^	1.50 (0.751, 3.00)	5.00 (1.01, 8.00)	2.500 (1.748, 3.751)^b^
t_½_, h	Mean (SD)	14.867 (2.277)	14.582 (1.873)	**…**	15.474 (2.652)	15.677 (2.505)	**…**
	Adjusted geomean	14.478	14.465	0.9991 (0.9635, 1.0360) [5.9]	15.263	15.492	1.0150 (0.9693, 1.0629) [7.4]
**Lamivudine**							
AUC_0‐∞_, h·µg/mL	Mean (SD)	12.930 (2.565)	13.068 (2.354)	**…**	14.883 (2.890)	13.603 (2.798)	**…**
	Adjusted geomean	13.436	12.867	0.9577 (0.9126, 1.0049) [7.8]	14.624	13.344	0.9114 (0.8658, 0.9593) [8.3]
AUC_0‐t_, h·µg/mL	Mean (SD)	12.783 (2.536)	12.850 (2.332)	**…**	14.715 (2.890)	13.351 (2.769)	**…**
	Adjusted geomean	13.282	12.649	0.9524 (0.9086, 0.9983) [7.6]	14.471	13.092	0.9048 (0.8592, 0.9528) [8.4]
C_max_, µg/mL	Mean (SD)	3.270 (0.906)	2.554 (0.478)	**…**	3.778 (1.241)	2.576 (0.895)	**…**
	Adjusted geomean	3.541	2.513	0.7097 (0.6474, 0.7779) [14.9]	3.582	2.445	0.6826 (0.5861, 0.7950) [25.0]
CL/F, L/h	Mean (SD)	24.112 (4.8134)	23.691 (4.451)	**…**	20.802 (3.617)	22.905 (4.515)	**…**
	Adjusted geomean	22.329	23.316	1.0442 (0.9951, 1.0957) [7.8]	20.489	22.481	1.0972 (1.0424, 1.1550) [8.3]
t_max,_ h	Median (min, max)	1.00 (0.500, 2.00)	3.50 (1.01, 5.00)	2.113 (1.500, 2.751)^b^	1.00 (0.503, 2.51)	2.75 (1.00, 6.00)	1.503 (0.998, 2.252)^b^
t_½_, h	Mean (SD)	18.014 (4.625)	19.918 (5.820)	**…**	18.697 (5.558)	20.152 (4.032)	**…**
	Adjusted geomean	18.415	19.118	1.0382 (0.8873, 1.2148) [26.6]	18.127	19.775	1.0909 (1.0012, 1.1887) [13.9]

AUC indicates area under the concentration‐time curve; CL/F, apparent clearance; C_max_, peak concentration; GLS, geometric least squares; %CV_w_, within‐subject coefficient of variation expressed as a percentage; PK, pharmacokinetic; t_max_, time to C_max_; t_½_, half‐time of elimination.

Note: The adjusted geometric mean is the anti‐log of the adjusted mean (ls‐mean) of log transformed values, where ls‐mean can be defined as a linear combination (sum) of the estimated effects from a linear model. GLS mean ratios were calculated as test (fed) vs reference (fasted). Fasted results are given for subjects administered formulation AH or AK after at least a 10‐hour fast, and fed results are given for subjects administered formulation AH or AK with a standard high‐fat meal.

a For part 1, the population number (n = 16) is representative of the subjects used to calculate the geometric mean for subject‐matched analysis. Arithmetic mean values for PK parameters were calculated from the total population of 76 subjects (n = 76).

b Value given as estimated median difference, calculated as Test – Reference (90% confidence interval of the difference).

### Adverse Events

Single‐dose administration of both formulations AH and AK was well tolerated. There were no deaths or serious AEs. Eighteen subjects (24%) had AEs with formulation AH, 12 subjects (16%) had AEs with formulation AK, and 29 subjects (19%) had AEs with the reference treatment (combination of subjects from part 1 and part 2). Most AEs were mild. Drug‐related AEs were similar across the treatment groups and in the fed versus fasted states (Table 4). Headache was the only drug‐related AE reported in more than 1% of subjects.

**Table 4:** Summary of Drug‐Related Adverse Events

	Reference	AH (fasted)	AK (fasted)	AH (fed)	AK (fed)
	N = 150	N = 76	N = 75	N = 16	N = 16
	n (%)	n (%)	n (%)	n (%)	n (%)
**Any event**	8 (5)	5 (7)	5 (7)	0	0
Headache	3 (2)	4 (5)	2 (3)	0	0
Dizziness	2 (1)	0	0	0	0
Diarrhea	1 (1)	0	0	0	0
Nausea	2 (1)	0	0	0	0
Vomiting	1 (1)	0	0	0	0
Fatigue	0	0	1 (1)	0	0
Abnormal dreams	0	1 (1)	0	0	0
Dyspepsia	0	0	1 (1)	0	0
Flatulence	0	0	1 (1)	0	0

Drug‐related adverse events are defined as adverse events that were considered related to study drug administration and are reported for the safety population. Reference treatment refers to coadministration of 50 mg dolutegravir and 300 mg lamivudine; it combines all subjects from part 1 and part 2. AH and AK represent subjects receiving either formulation AH or AK in the fasted state (after at least a 10‐hour fast), and fed represent subjects receiving either formulation AH or AK after a standard high‐fat meal. Values are given as number of events (percentage of the population).

Two subjects in part 1 and 1 subject in part 2 had AEs leading to discontinuation and withdrawal. In part 1, 1 subject experienced a grade 1 ALT elevation (96 IU/L; normal range 21 to 72 IU/L), which was deemed not related to study drug (formulation AH). The ALT value returned to normal by study day 21. Also in part 1, 1 subject experienced grade 1 vomiting that occurred within 4 hours of formulation AH administration and that was related to intravenous catheter insertion. In part 2, 1 subject experienced grade 1 creatinine elevation (132.6 µmol/L; normal range 44.2 to 114.9 µmol/L) on day 7 after receiving a single dose of formulation AK. This AE was due to the use of creatine and protein supplements and was deemed unrelated to study drug.

There were no trends in clinical laboratory evaluations, vital signs, or hematology parameters. ECG findings were either normal or were abnormal (outside of reference ranges) but not considered clinically significant.

## Discussion

Two‐drug, fixed‐dose combination tablets have the potential to decrease cumulative drug exposure, and perhaps toxicity, as compared with 3‐drug regimens. Streamlined treatment regimens, especially single‐tablet regimens, may improve treatment compliance. Because coadministration of single‐entity tablets of dolutegravir and lamivudine were recently demonstrated to be well tolerated and effective for treating HIV‐1,^25^ the objective of the current study was to evaluate the bioequivalence of 2 novel dolutegravir and lamivudine (50 mg and 300 mg, respectively) fixed‐dose combination tablet formulations (AH and AK), using coadministration of 50 mg dolutegravir and 300 mg lamivudine tablets as the reference treatment. Safety and the potential effects of food were also evaluated.

Formulation AH exhibited lamivudine exposure that was similar to lamivudine exposure after coadministration of the single‐entity tablets, as AUC_0‐∞_, AUC_0‐t_, and C_max_ GLS mean ratios and 90%CI values conformed to bioequivalence standards. However, dolutegravir exposure was consistently higher than the reference, and t_max_ was similar to the reference (Table 2), indicating a higher rate and extent of absorption of dolutegravir from formulation AH. Bioequivalence for this compound was therefore not achieved.

Formulation AK exhibited lamivudine AUC_0‐∞_ and AUC_0‐t_ values similar to the reference; however, C_max_ was approximately 32% higher. Dolutegravir exposure was similar to the reference as measured by AUC_0‐∞_ and C_max_. However, AUC_0‐t_ was ∼16% higher in the test formulation, and the upper 90%CI limit of the AUC_0‐t_ GLS means ratio slightly exceeded the 1.2500 bioequivalence threshold, with a value of 1.2507. However, differences in test/reference AUC_0‐t_ time windows may cause sufficient bias in the AUC_0‐t_ geometric mean ratios to influence the determination of bioequivalence.^30^ A sensitivity analysis, which excluded 2 subjects with between‐treatment differences in t_last_, indicated that formulation AK was bioequivalent to the reference. Therefore, dolutegravir exposure from formulation AK can be considered bioequivalent to the reference.

The higher lamivudine C_max_ for formulation AK despite similar t_max_ values to the reference (Table 2) suggests a faster lamivudine absorption rate; however, potential reasons for this absorption change are unclear. Lamivudine is highly soluble over the physiologic pH range,^31^ and bioenhancement has not been observed. In addition, formulation AK has rapid and complete dissolution in vitro within 10 minutes (data not shown), and a previous relative bioavailability clinical study of the single‐entity lamivudine demonstrated that a 100‐mg capsule, a 100‐mg tablet, and a 1 mg/mL alcohol‐free oral solution were bioequivalent for AUC and C_max_.^20^ Last, lamivudine permeability in Caco‐2 cells was similar in both the presence and absence of dolutegravir (data not shown). These results indicate that disintegration and dissolution are not rate‐limiting factors for lamivudine absorption and permeability effects also do not explain observations of elevated C_max_ in formulation AK.

Although the underlying mechanism of faster lamivudine absorption rate remains unknown, the higher lamivudine C_max_ is not considered clinically significant. Lamivudine AUC is considered the best plasma pharmacokinetic predictor of efficacy,^32^ and AUC values were bioequivalent (Table 2). Additionally, long‐term (24 weeks) dose ranging studies with lamivudine^24^, ^25^ have not indicated a dose‐related increased risk of AEs or laboratory abnormalities with doses up to 840 mg daily for a 70‐kg subject. Finally, multiple phase 3 studies have failed to demonstrate any dose‐related increases in the overall incidence of AEs, and there were no significant safety or efficacy differences between the standard lamivudine 300 mg/d regimen and a higher 600 mg/d regimen.^33^, ^34^, ^35^

A food effect for both dolutegravir and lamivudine exposure parameters was noted (Table 3), with administration of a high‐fat meal increasing dolutegravir AUC for formulation AH by ∼15% and for formulation AK by ∼32%, increasing dolutegravir C_max_ for formulation AK by ∼21%, and reducing lamivudine C_max_ for both formulations by ∼30%. These increases in dolutegravir AUC and C_max_ and decreases in lamivudine C_max_ in response to food are consistent with the known effect of food on the individual components,^18^, ^36^, ^37^ which is not expected to impact safety or efficacy.^32^, ^38^

Single doses of the experimental formulations were well tolerated. Most subjects did not report any AEs, and most of the reported AEs were mild. The drug‐related AEs were consistent with the known profiles of dolutegravir and lamivudine. Headache, a known common side effect of both drugs, was the only drug‐related adverse event that occurred in more than 1% of the study population (3% to 5%). These results support the safety and tolerability of the combination formulations.

In conclusion, although single doses of both formulations AH and AK were safe and well tolerated, the pharmacokinetic data demonstrate that formulation AK is most comparable to the treatment regimen used in the GEMINI studies. Formulation AK met bioequivalence standards for dolutegravir AUC and C_max_ and for lamivudine AUC when compared with coadministration of 50‐mg dolutegravir tablets and 300‐mg lamivudine tablets. A higher lamivudine C_max_, which reflects differences in the rate but not the extent of absorption, was observed for formulation AK but is not expected to significantly affect patient safety or antiviral efficacy based on historical data. Taken together, the data from this bioequivalence and food effect study provide an acceptable pharmacokinetic bridge between the formulation AK fixed‐dose combination tablet and the single‐drug entities used in the GEMINI‐1 and GEMINI‐2 studies. These results supported further development of formulation AK, which was recently approved in the United States and the European Union as Dovato, a 2‐drug, fixed‐dose combination therapy for the treatment of patients with HIV‐1.
